# Erdheim-Chester Disease Presenting as a Suprasellar Mass

**DOI:** 10.7759/cureus.109622

**Published:** 2026-05-25

**Authors:** Anshu Bandhlish, Gabrielle A White-Dzuro, Ariana Barkley, Brian Hanak, Caitlin S Latimer, Michelle M Cecchini, Luis Gonzalez-Cuyar, Manny Ferreira

**Affiliations:** 1 Laboratory Medicine and Pathology, University of Washington School of Medicine, Seattle, USA; 2 Anesthesiology and Critical Care, Massachusetts General Hospital, Boston, USA; 3 Neurological Surgery, School of Medicine, University of Colorado, Boulder, USA; 4 Neurological Surgery, Children’s Hospital of Orange County, Orange, USA; 5 Neurological Surgery, Texas Neuro Spine, San Antonio, USA; 6 Laboratory Medicine and Pathology, University of Washington Medical Center, Seattle, USA; 7 Neurological Surgery, University of Washington School of Medicine, Seattle, USA

**Keywords:** braf mutation, diabetes insipidus, erdheim-chester disease, non-langerhans cell histiocytosis, suprasellar mass

## Abstract

Erdheim-Chester disease (ECD) is a rare, sporadic, non-Langerhans cell histiocytosis with multiorgan involvement. Less than half of patients present with central nervous system (CNS) involvement, usually affecting the hypothalamic-pituitary axis (HPA), meninges, and brain parenchyma. CNS-only involvement is extremely rare and can lead to challenges in both diagnosis and management. Clinical course of the disease is varied, ranging from indolent to disabling and life-threatening forms. We report a case of a 55-year-old woman who presented with panhypopituitarism and central diabetes insipidus (DI) in 2016 and was found to have a rapidly enlarging suprasellar mass with non-specific radiologic features. Transcortical biopsy at the time of presentation was inconclusive, and high-dose corticosteroid therapy with close clinical follow-up was initiated. Over a period of six months, the patient had progressive disease with involvement of the hypothalamus and profound tracking cytotoxic edema. Surgical management in the form of endonasal, extended transsphenoidal resection was performed. Final pathology demonstrated a histiocytic neoplasm positive for BRAF V600E mutation (VE1) by immunohistochemistry, consistent with ECD. This case highlights the diagnostic challenges associated with ECD involving the CNS, particularly in anatomically complex regions where tissue sampling may be limited and histologic findings non-specific. While BRAF-targeted therapies were not routinely available at the time of the patient’s presentation, identification of BRAF mutations has significant therapeutic implications, enabling the use of targeted therapies in patients with unresectable or progressive disease. This case underscores the importance of maintaining a high index of suspicion in patients presenting with central DI with indeterminate suprasellar lesions.

## Introduction

Erdheim-Chester disease (ECD) is a rare, non-Langerhans histiocytic disorder that was first described in 1930 [1]. It is characterized by infiltration of tissues with CD68-positive, CD1a-negative histiocytes. Patients commonly present with bone pain, diabetes insipidus, fever, or other systemic symptoms. While it frequently involves multiple organ systems, central nervous system (CNS) involvement has been reported in approximately 25-50% of cases [2]. Neurologic symptoms at presentation can include headaches, cerebellar signs, mental status changes, and seizures. When the CNS is involved, it is typically found within the hypothalamus, brainstem, and cerebellum. CNS involvement is the sole independent clinical predictor of mortality and directly accounts for nearly a third of deaths among patients with ECD [3,4].

While consensus guidelines have been created for the diagnosis and management of ECD, the rarity with which most physicians encounter this disease makes its treatment difficult [5]. ECD is a clonal neoplasm that involves mutation of the BRAF V600E gene, identified in around 65% of cases. Targeted therapies, such as BRAF and MEK inhibitors, have emerged as a preferred treatment in patients with ECD, especially in cases of CNS/cardiovascular involvement and refractory cases of ECD. These therapies have robust overall efficacy, with no acquired resistance reported to date [4].

We present a case of BRAF-altered ECD in a patient presenting with diabetes insipidus (DI) and an aggressively expansile suprasellar mass who died nine months after initial presentation. This report highlights diagnostic challenges associated with histiocytic neoplasms involving the CNS due to their non-specific clinical, radiographic, and histopathological findings. A high level of suspicion and awareness of this rare diagnosis is essential to avoid delayed diagnosis and treatment.

## Case presentation

We present a case of a 55-year-old woman who presented to the emergency department (ED) with lower extremity edema and altered mental status, prompting non-contrast head computed tomography (CT), which revealed a suprasellar mass with non-specific radiological features (Figure 1). Brain magnetic resonance imaging (MRI) with and without contrast showed a 2.5x2.5x1.9 cm enhancing suprasellar mass (Figures 2A-2D). On further work-up, the patient was found to have central DI and panhypopituitarism. Chest CT performed at initial presentation showed only two small pulmonary nodules of indeterminate significance. Visual field examination showed a dense inferotemporal visual field loss. Radiologically, the differential diagnosis was broad and included an optic nerve glioma, teratoma, craniopharyngioma, hypothalamic glioma, lymphoma, sarcoidosis, meningioma, and metastases, among others. The patient was taken to the operating room for right frontotemporal craniotomy with posterolateral orbitotomy for biopsy of the mass. Histopathological analysis was inconclusive at the time, revealing fibrovascular tissue involved by reactive inflammatory infiltrates predominantly composed of macrophages, lymphocytes, and plasma cells. The neuropathologic differential diagnosis at that time included a reactive histiocytic proliferation, sarcoidosis, lymphocytic hypophysitis, and an infectious process, among others.

**Figure 1:**
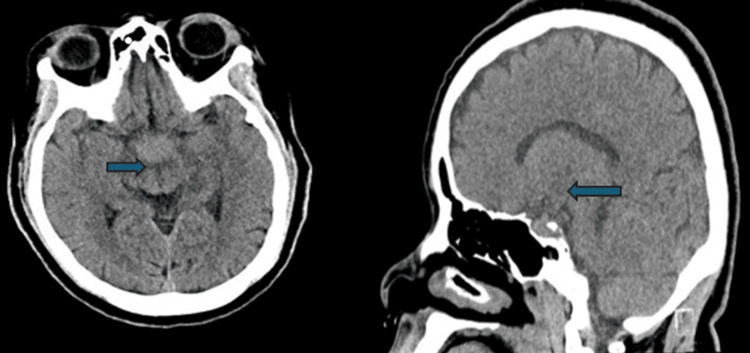
Preoperative CT images (axial and sagittal cuts) demonstrate hyperdensity within the hypothalamus (arrows).

**Figure 2:**
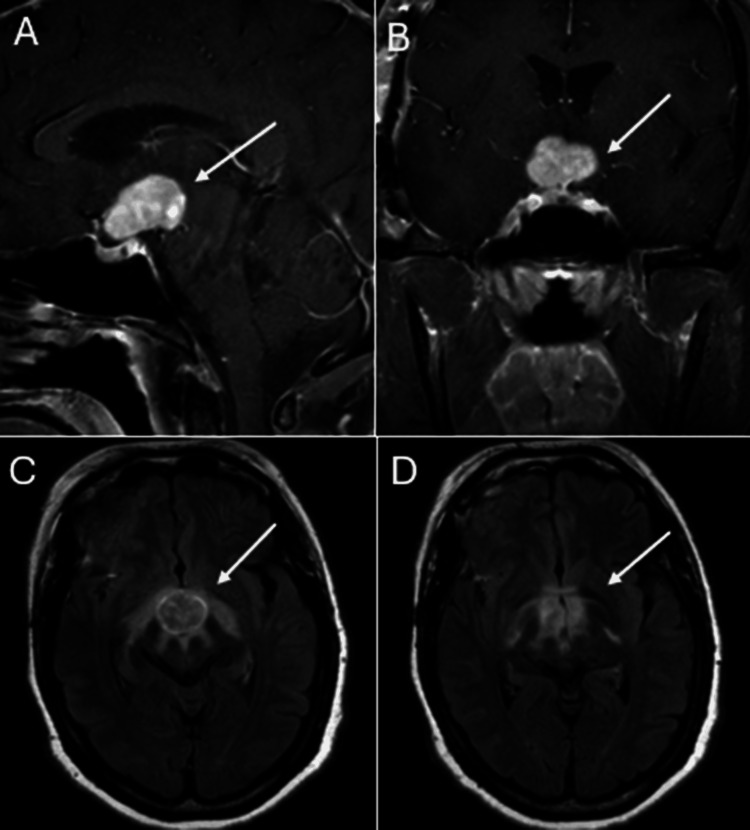
Magnetic resonance imaging (MRI). Preoperative images - sagittal and coronal cuts of contrasted T1-weighted MRI showing a 2.5 cm midline suprasellar mass (arrows) (A, B). The mass demonstrates robust enhancement and is isointense in T1- and T2-weighted images. Axial FLAIR MRI showing edema surrounding the mass (arrows) (C, D). FLAIR: fluid-attenuated inversion recovery

As an outpatient, the patient was treated with a short course of steroids, and her panhypopituitarism was managed with hormone replacement therapy. A diagnostic lumbar puncture was also performed, and cerebrospinal fluid (CSF) analysis demonstrated albuminocytologic dissociation (ACD) with lymphocytosis. CSF cultures were negative, and blood angiotensin converting enzyme (ACE) levels were normal at 19 U/L (reference range: 16-85 U/L). There was no evidence of ocular sarcoidosis. Over the course of five months, the lesion increased in size with a significant amount of edema surrounding the hypothalamus, bilateral optic tracts, optic nerves, and cerebral peduncles on MRI (Figures 2A-2D). Subsequently, the patient appeared to have deteriorated significantly with persistent altered mental status, acute DI, and significant fatigue. Given the clinical and radiological progression, a repeat biopsy was recommended for a more definitive diagnosis.

The patient was scheduled for surgery, but just days before the planned date, presented to the ED with altered mental status, in acute DI with a sodium level of 157 mEq/L (reference range: 135-145 mEq/L). After medical stabilization in consultation with endocrinology consultants, the patient underwent endoscopic endonasal transsphenoidal biopsy with placement of right frontal external ventricular drain (EVD).

Histopathological examination demonstrated fragments of gliotic neural parenchyma involved by a mixed population of histiocytic cells in a background of mixed inflammatory cells, including lymphocytes, plasma cells, and occasional eosinophils. The histiocytic cells were characterized by abundant eosinophilic cytoplasm and eccentrically placed ovoid nuclei with vesicular chromatin and variably prominent nucleoli. No emperipolesis was identified. While multinucleated giant cells were present, well-formed granulomas were not identified (Figures 3A, 3B). Neuropathological differential diagnosis was broad, including reactive histiocytic proliferation; histiocytic neoplasms such as Langerhans cell histiocytosis (LCH), extranodal Rosai-Dorfman disease (RDD), ECD, and ALK-positive histiocytosis; infection; IgG4-related disease; lymphoma; and granulomatous inflammation. On immunohistochemical staining, the histiocytic cells were positive for CD68 and CD163 (Figure 3C) and for BRAF (V600E) (Figure 3E), and negative for S100 (Figure 3D), CD1a (Figure 3F), langerin, and ALK (D5F3). Immunohistochemical stains also showed a mixed population of lymphoid cells consisting of CD3-positive T-cells, which were more numerous than CD20-positive B-cells. The plasma cells were highlighted by CD138, with rare IgG4-positive plasma cells and an IgG4/IgG ratio that was within normal limits. Special stains for mycobacterial, fungal, bacterial, and spirochetal organisms were negative. Intraoperative microbiological cultures and PCR studies were also negative for microorganisms.

**Figure 3:**
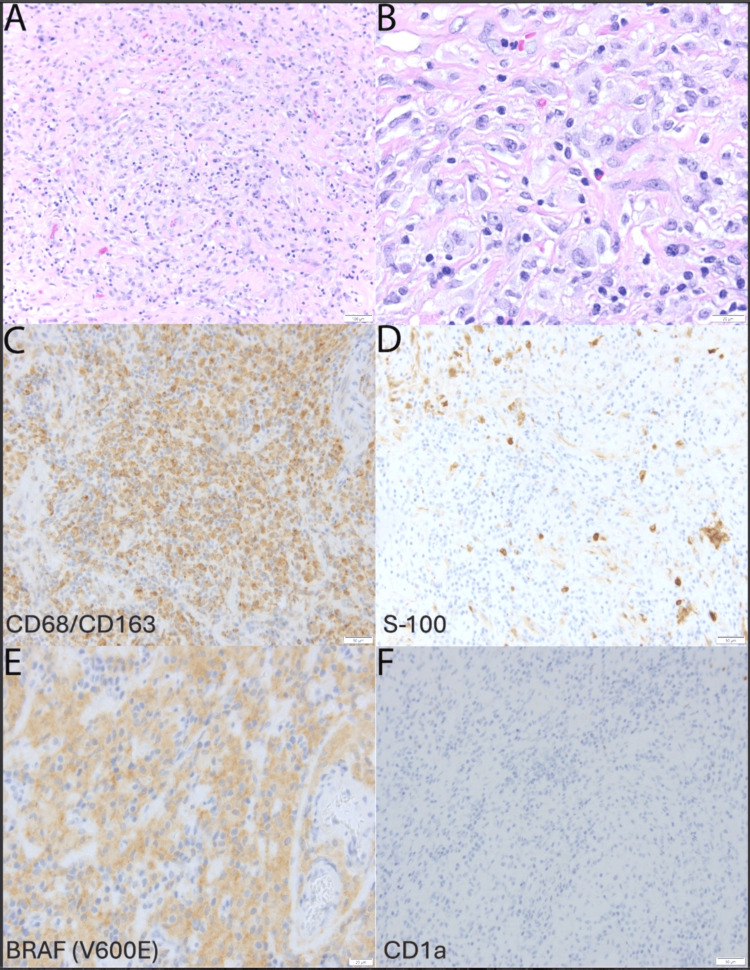
Histologic and immunohistochemical features. A (10×) and B (40×) hematoxylin and eosin-stained sections demonstrate fragments of gliotic neural parenchyma involved by a mixed population of histiocytic cells in a background of mixed inflammatory cells, including lymphocytes, plasma cells, and occasional eosinophils. The histiocytic cells are characterized by abundant eosinophilic cytoplasm and eccentrically placed ovoid nuclei with vesicular chromatin and variably prominent nucleoli. No emperipolesis is identified. On immunohistochemistry, the histiocytic cells are positive for CD68 and CD163, C (20×), and BRAF (V600E), E (40×), and negative for S100, D (20×), and CD1a, F (20×).

The final pathologic diagnosis was determined to be histiocytosis with a BRAF V600E mutation consistent with ECD. Follow-up imaging showed residual tumor in the suprasellar cistern and infundibulum. The patient continued to deteriorate clinically and died of the disease nine months after the time of first clinical presentation.

## Discussion

ECD was first described by William Chester and the Austrian pathologist Jacob Erdheim in 1930 [1]. ECD is a rare inflammatory myeloid clonal disease classified within the histiocytosis. MAPK-ERK pathway-activating mutations are found in over 80% of cases, with the BRAF V600E mutation predominant [4,6]. Diagnosis of ECD is challenging owing to a wide range of clinical, morphologic, and radiological presentations [4,7-9].

ECD mainly affects adults aged 50-60 years, with an average age at presentation of 53 years. There is a slight male predominance (2-3:1) with only a few documented cases in the pediatric population [3]. ECD manifests as a systemic disease with multiorgan involvement, with the most common sites of involvement being the bones, kidneys, and central nervous system, with additional sites including the orbit, mediastinum, skin, and retroperitoneum, as well as the endocrine, pulmonary, and cardiovascular systems. The most frequent clinical presentation is bone involvement, in the form of long bone osteosclerosis, seen in 80-95% of cases, with bone pain in 38% of cases [4]. Neurological involvement as the initial presentation of ECD is rare but can be seen in up to 25% of cases [10-12]. As with our case, the most frequent presenting neurological sign is DI of “unknown origin” (16%), followed by other neurological symptoms (14%) [13]. The most frequent neurological presentations are cerebellar (41%) and pyramidal syndromes (45%); however, seizures, sensory disturbances, headaches, cognitive impairment, cranial nerve palsies, and even asymptomatic lesions have been reported [14]. Multifocality of the disease precludes surgical resection as a viable curative option. Surgery is recommended in cases with tumors involving vital organs (CNS, ocular) for tumor debulking and immediate palliation of symptoms [5].

Radiologically, neurological involvement of ECD may have a tumoral or degenerative presentation. On MRI, CNS manifestations may appear as infiltrative, enhancing parenchymal lesions with widespread nodules involving the brainstem, cerebrum, cerebellum, hypothalamus, diencephalon, and pituitary [4]. The infiltrative lesions demonstrate increased signal intensity on T2- and fluid-attenuated inversion recovery (FLAIR)-weighted images, homogeneous hypointensity on T1-weighted imaging, and homogeneous enhancement on postcontrast imaging [15]. Meningeal pattern, in the form of diffuse thickening of the dura, can be seen in 19-23% of cases [15]. Based on imaging findings, the differential diagnosis often includes multiple sclerosis, primary intracranial neoplasms, sarcoidosis, and histiocytic disorders, among others.

Multimodal imaging modalities have been utilized to comprehensively evaluate the extent of disease, guide biopsies, and gauge treatment response. Computed tomography (CT), magnetic resonance imaging (MRI), 99mTc methylene diphosphonate scintigraphy (bone scan), and fluorodeoxyglucose (FDG) positron emission tomography (PET)/CT scans thus play a key role in the diagnosis and management of the disease. Symmetric diaphyseal and metaphyseal osteosclerosis of the lower extremities, one of the most common findings in ECD, can be identified on CT, PET, and bone scans [4]. A PET scan is considered superior to a bone scan because it evaluates not only the extent of the disease but also its activity. In a study by Young et al., 71 18F-FDG PET/CT scans were performed in 32 patients with a confirmed diagnosis of ECD over a period of 10 years. In their study, BRAF mutation was associated with FDG-avid CNS disease (p=0.0357), higher SUVmax (p=0.0044), and greater mortality (p=0.0215) [16].

While CNS ECD is commonly located in regions not amenable to surgical excision, a biopsy from the most accessible location should be attempted to confirm the diagnosis and establish BRAF mutational status. Techniques such as droplet digital polymerase chain reaction (PCR) and next-generation sequencing (NGS) are utilized to explore the genetic landscape of ECD, including BRAF and other mutations in the MAPK pathway [17]. Ethylenediaminetetraacetic acid (EDTA)-based decalcification methods should be employed in bone biopsy cases to aid nucleic acid preservation for molecular and biomarker testing [18]. CSF sampling can be performed to exclude other etiologies such as infection, malignancy, and sarcoidosis. CSF analysis is commonly negative for any abnormalities or may demonstrate a possible increase in protein and/or cellularity.

Tumor-derived cell-free DNA (cfDNA) can be detected in plasma, CSF, and urine; thus, liquid biopsy samples (plasma, CSF, and urine) can serve as an alternative source of biological material for testing in complex cases. BRAF V600E mutational testing using a plasma sample has shown high overall accuracy compared with a paired tissue sample across multiple studies [19]. Hence, circulating cfDNA is an attractive option for evaluating mutational status, including in cases where a biopsy is difficult to obtain or initially inconclusive, as was seen in our case. Larger prospective studies are needed to further validate the accuracy of cfDNA for early detection of ECD.

Since the patient’s presentation in 2016, most patients with ECD have required systemic therapy at the time of diagnosis. Interferon-α-2a and pegylated interferon-α-2a are the more conventional therapies used in ECD, with response rates of 50-80% and approximately 64% in CNS cases [20]. Cytokine-directed therapy, including interleukin-1 (IL-1) receptor antagonists (anakinra, canakinumab), IL-6 receptor antagonists (tocilizumab), and tumor necrosis factor-α (TNF-α) inhibitors (infliximab, etanercept), has been evaluated as therapeutic agents, but their responses have been inconsistent in CNS disease [21]. The role of chemotherapeutic agents is limited, with cladribine being the most commonly used. Identification of the pivotal role of the mitogen-activated protein kinase (MAPK) pathway, found in up to 80% of cases of ECD, has revolutionized research and therapeutic options for ECD. BRAF inhibitors such as vemurafenib or dabrafenib are now considered the first-line therapy in patients with life-threatening neurological involvement [22].

## Conclusions

We report a case of BRAF V600E-mutant ECD presenting as a suprasellar mass with DI, panhypopituitarism, and visual field deficits. This case underscores the importance of considering ECD in the differential diagnosis of indeterminate CNS lesions, particularly in patients with focal CNS lesions of unclear etiology. Early recognition, repeat tissue sampling when necessary, and timely molecular characterization are critical to establishing the diagnosis. In challenging cases where tissue is limited or inaccessible, liquid biopsy approaches using circulating tumor-derived cell-free DNA may provide a valuable adjunct for detecting actionable mutations. Identification of BRAF mutations is especially impactful, as it enables the use of targeted therapies that may significantly alter the disease course in patients with otherwise progressive and life-threatening CNS involvement.
